# Pancratistatin induces apoptosis in clinical leukemia samples with minimal effect on non-cancerous peripheral blood mononuclear cells

**DOI:** 10.1186/1475-2867-10-6

**Published:** 2010-03-06

**Authors:** Carly Griffin, Caroline Hamm, James McNulty, Siyaram Pandey

**Affiliations:** 1 Department of Chemistry & Biochemistry, University of Windsor, Windsor, ON Canada; 2 Windsor Regional Cancer Center, Windsor, ON Canada; 3 Department of Chemistry, McMaster University, Hamilton, ON Canada

## Abstract

**Background:**

Pancratistatin, a natural compound extracted from *Hymenocallis littoralis*, can selectively induce apoptosis in several cancer cell lines. In this *ex vivo *study, we evaluated the effect of pancratistatin on peripheral blood mononuclear cells obtained from 15 leukemia patients prior to clinical intervention of newly diagnosed patients, as well as others of different ages in relapse and at various disease progression states.

**Results:**

Mononuclear cells from healthy volunteers and leukemia patients were exposed to 1 μM pancratistatin for up to 48 h. Irrespective of leukemia type, pancratistatin induced apoptosis in the leukemic samples, with minimal effects on non-cancerous peripheral blood mononuclear control cells.

**Conclusion:**

Our results show that pancratistatin is an effective and selective anti-cancer agent with potential for advancement to clinical trials.

## Background

Chemotherapy for acute myeloid leukemia, despite earnest attempts, has not significantly changed in the last 30 years. Treatment continues to be based on the cytotoxic chemotherapies of anthracyclines and cytarabine. The natural compound, pancratistatin, extracted from the *Hymenocallis littoralis*, has broad-range efficacy against several cancer cell lines at 1 μM, with minimal effect on non-cancerous cell lines of the same origin [1,2]. Pancratistatin treatment causes phosphatidyl-serine flipping, caspase-3 activation, generation of reactive oxygen species (ROS), and loss of mitochondrial membrane potential, which leads to apoptosis in cultured T-cell (Jurkat) leukemia cells [3]. Although pancratistatin is a non-genotoxic drug, its target has not yet been elucidated [3,4]. The efficacy of pancratistatin in inducing apoptosis selectively in cultured (commercial) cancer cell lines is well established, though its effect on leukemia cells obtained from patients has not been tested. In this study, clinical leukemia and non-cancerous peripheral blood mononuclear cells (ncPBMCs) were treated with pancratistatin to determine its selectivity and efficacy to induce apoptosis *ex vivo*. Activity of pancratistatin was compared to that of the widely used chemotherapeutic, paclitaxel, against cancer cells. Peripheral blood samples from patients with a diagnosis of acute myeloid leukemia (AML; n = 11), acute lymphoid leukemia (ALL; n = 1), chronic myelogenous leukemia (CML; n = 1), chronic myelomonocytic leukemia (CMML; n = 1) and Mantle cell lymphoma (n = 1) were obtained. The majority of samples were taken at diagnosis, that is, in chemo-näive patients. Of the 15 patients, 5 did not go into remission with induction chemotherapy. The median duration of remission was 3 months. Our pre-clinical results demonstrate that pancratistatin is effective against all types of leukemia tested and does not induce apoptosis in non-cancerous mononuclear cells.

## Methods

### Cell Culture

Human T-cell (Jurkat) leukemia cells were purchased from ATCC and maintained in RPMI 1640 medium supplemented with 10% fetal bovine serum and 10 μM gentamycin in an incubator set at 37°C and 5% CO_2 _in air. Peripheral blood was obtained from leukemia patients at the Windsor Regional Cancer Center (WRCC, Windsor Regional Hospital REB #04-043 and 04-044) upon written, informed consent and from healthy non-smoking volunteers aged 25-50 y (University of Windsor REB #04-147). Whole blood samples were collected in BD Vacutainer™ Cell Preparation Tubes, and mononuclear cells were separated by density gradient centrifugation. The isolated cells were maintained in RPMI 1640 media supplemented and maintained in the same way as the Jurkat cultures. Cells were treated within 3 h of collection with 1 μM pancratistatin or 500 nM paclitaxel for up to 48 h.

### Apoptosis Assays

Apoptotic cells were detected by microscopy with cell-permeable Hoechst 33342 dye (Molecular Probes, Eugene OR) or by flow cytometry with Annexin-V AlexaFluor488 (Sigma-Aldrich, Missisauga, ON) using established protocols [1]. Briefly, cells were treated with 1 μM pancratistatin for either 24 or 48 h, or with 500 nM paclitaxel for 24 h. For flow cytometry, cells were washed twice in room temperature PBS, resuspended in Annexin-V binding buffer and incubated with AlexaFluor-488 (1:50) for 15 min. They were washed in calcium-binding buffer and a minimum of 20,000 were analyzed on a Beckman Coulter Cytomics FC500 flow cytometer. To achieve the highest cell number per sample, propidium idodide was not used in this study. For microscopy, 5 min incubation with 10 μM Hoechst 33342 dye was added to the Annexin-V binding step. Brightly stained, condensed nuclei were visible with Hoechst dye, characteristic of apoptotic cells, as is phosphatidylserine externalization detected by Annexin-V binding. Images were captured on an inverted fluorescence microscope (Leica DM IRB, Germany). The percentage of apoptotic cells was calculated from a minimum of 5 fields with >100 cells/field. Statistical analysis included the unpaired t-test, and two-tailed p value; † represents p < 0.05 between means of untreated and pancratistatin treated leukemia samples, which was taken as statistically significant.

## Results

### Pancratistatin induces apoptosis in cultured leukemia cells

**Figure 1:**
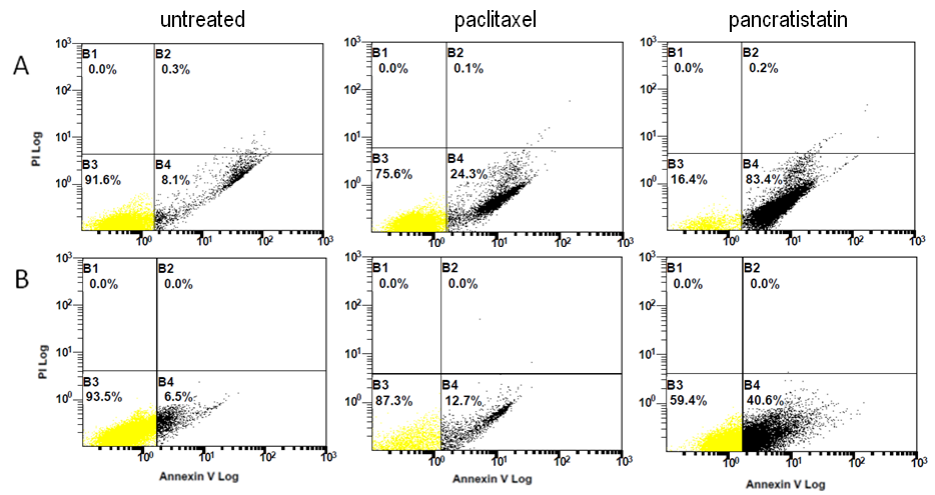
**Detection of apoptosis in cells treated for 24 h with paclitaxel or pancratistatin**. Flow cytometry with Annexin-V AlexaFluor 488 was used to detect apoptosis in Jurkat cells (A) and patient-obtained leukemic PBMCs (B) that were untreated, treated with 500 nM paclitaxel or treated with 1 μM pancratistatin for 24 h. The leukemic PBMCs were obtained from a chemo-näive female patient (65 y) diagnosed with AML -- M1. Flipping of phosphatidylserine from the inner to the outer leaflet of the plasma membrane, which binds to Annexin-V in the presence of calcium, is a characteristic feature of apoptosis. A minimum of 20 000 events were measured for each sample on a Beckman Coulter Cytomics FC500 flow cytometer.

From previous experiments on the effects of pancratistatin on cultured Jurkat cells (T-cell leukemia), the majority of cells were apoptotic after 24 h [3]. Pancratistatin rapidly induced apoptosis in >80% of Jurkat cells in the present study, whereas paclitaxel induced cell death in ~25% of cells, as determined by flow cytometry using Annexin-V AlexaFluor488 (Figure 1A).

### Pancratistatin induces apoptosis in clinical leukemia samples

**Figure 2:**
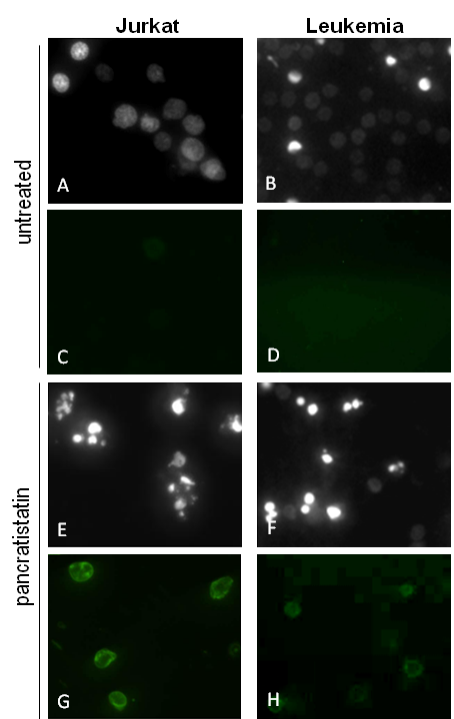
**Response of clinical leukemia and cultured Jurkat cells to treatment with pancratistatin**. The apoptotic effect of 24 h exposure to 1 μM pancratistatin was observed by microscopy using cell-permeable Hoechst dye. Compared to the nuclear morphology of untreated cells (A, B), treatment with pancratistatin resulted in apoptosis characterized by condensed, brightly stained nuclei in Jurkat (E) and patient obtained leukemic PBMCs (F). Annexin-V binding, another characteristic feature of apoptosis, was minimal in untreated cells (C, D) in contrast to the binding observed after pancratistatin treatment (G, H). Hoechst and Annexin-V images are not of the same field.

Table 1 gives details of each patient's age, leukemia type and percent blasts at diagnosis, alongside their response to pancratistatin treatment *ex vivo*. Apoptosis percentage values are corrected for the percentage of apoptosis observed in untreated samples of the same patient. All diagnoses are 1^st ^occurrence unless otherwise stated. Patients were diagnosed in the clinic with: Acute Myeloid Leukemia (AML), subtyped as M0 - M7 or MDS (myelodysplastic syndrome); Acute Monocytic Leukemia (AMoL); Acute Lymphoblastic Leukemia (ALL), subtyped as Burkitt's (L3); Chronic Myelogenous Leukemia (CML); or Chronic Myelomonocytic Leukemia (CMML). Response to chemotherapy administered in the clinic is indicated as complete response (CR), partial response (PR), no response (NR), relapsed or censored. Nine of the 15 samples showed an increase in apoptosis of 40% greater than untreated PBMCs of the same patient after exposure to pancratistatin, as determined by Hoechst staining (Table 1). This result was confirmed by detection of Annexin-V AlexaFluor488 binding by flow cytometry (Figure 1B). Figure 2 shows the efficacy of pancratistatin on both cultured leukemia (Jurkat) cells and patient-obtained leukemia PBMCs as observed by Hoechst staining and Annexin-V binding.

**Table 1:** Clinical features, disease state, response in the clinic and *in vitro *of patient-obtained leukemia treated with pancratistatin.

Patient #	Age	Clinical Diagnosis	% Blasts	Clinical Response	Exposure	% Apoptosis
1	60	AML - M3	85%	censored	24 h	64.9 ± 4.2

2	81	AML-M2	20%	NR	24 h	33.5 ± 7.7

3	53	AMoL-M5a	96%	CR	24 h	46.5 ± 7.9

4	73	CML	9%	censored	24 h	63.9 ± 2.2

5	23	AML-M5a	70%	NR	24 h	30.6 ± 1.6

6	66	ALL-L3	34%	NR	48 h	61.8 ± 9.8

7	62	CMML-M4	32%	NR	48 h	17.7 ± 2.7

8	74	AML-M2	60%	NR	48 h	48.6 ± 6.7

9	45	AML	84%	censored	48 h	63.9 ± 4.2

10	57	AML-M3	43%	PR	48 h	70.8 ± 5.9

11	68	AML-M2	n/a	CR	48 h	46.8 ± 12.2

12	80	Mantel cell lymphoma	0%	censored	48 h	35.2 ± 6.6

13	47	AML	44%	censored	48 h	37.3 ± 7.7

14	71	AML-M0, relapsed	52%	relapsed	48 h	45.3 ± 11.3

15	65	AML-MDS, relapsed	48%	CR	48 h	37.3 ± 8.5

### Pancratistatin is non-toxic to non-cancerous PBMCs

**Figure 3:**
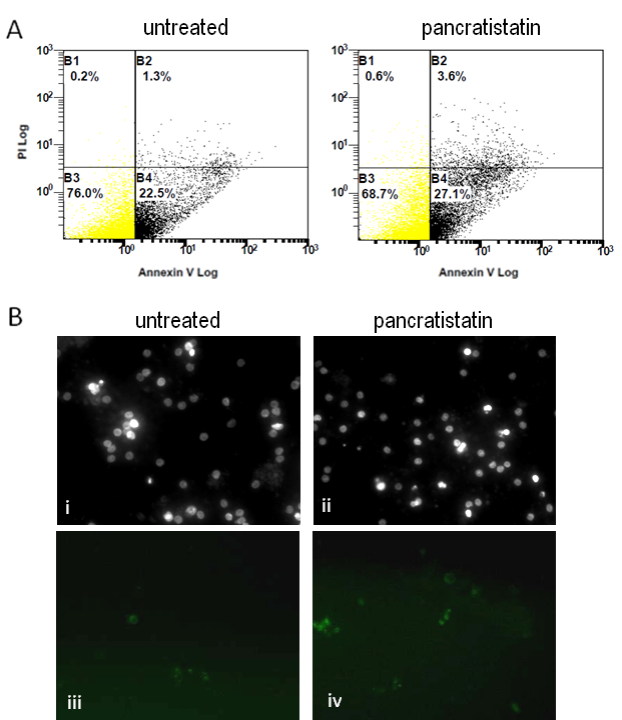
**Non-cancerous peripheral blood mono-nucleated cells (PBMCs) are relatively unaffected by pancratistatin**. A) Flow cytometry analysis using Annexin-V AlexaFluor 488 was performed on PBMCs that were untreated or treated with 1 μM pancratistatin for 48 h. A minimum of 20 000 events were measured for each sample on a Beckman Coulter Cytomics FC500 flow cytometer. B) Hoechst staining and Annexin-V binding depicts the selective activity of pancratistatin; there is minimal difference in nuclear morphology (i, ii) and amount of externalized phosphatidylserine (iii, iv) between untreated and treated non-cancerous PBMCs.

**Figure 4:**
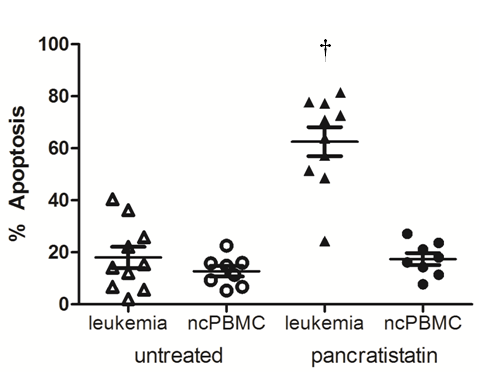
**Comparative effect of 48 h treatment with pancratistatin**. Leukemia samples (n = 10) and non-cancerous PBMCs (n = 8) treated for 48 h with pancratistatin were stained with Hoechst dye and counted manually (a minimum of 5 fields with 100 cells per field); percent apoptosis was calculated per total cell number. Statistical analysis was performed (unpaired t-test, two-tailed p value); † represents p < 0.05 between untreated and pancratistatin treated leukemia samples.

To determine if the apoptosis-inducing effect of pancratistatin is selective for cancer cells, PBMCs isolated from blood samples from healthy volunteers (n = 8) were treated with 1 μM pancratistatin for up to 48 h. Interestingly, pancratistatin did not enhance apoptosis in these cells as in leukemic PBMC isolates, as determined by flow cytometry analysis using Annexin-V binding and nuclear morphology after Hoechst staining (Figure 3A, B). The cancer-selective apoptosis-inducing activity of pancratistatin was a trend that was observed in all samples obtained (Figure 4). This data demonstrates that pancratistatin is selectively toxic to cancer cells *ex vivo*, irrespective of leukemia type, with an insignificant effect on non-cancerous PBMC isolates.

## Discussion

We report the novel finding that pancratistatin has selective apoptosis-inducing activity *ex vivo *at a low dose in a random sampling (n = 15) of patient-obtained leukemias. In contrast, pancratistatin has no effect on non-cancerous peripheral blood mononuclear cells (n = 8). Cellular subtypes in the PBMC samples may have differing sensitivities to pancratistatin and separation of these populations is needed to clarify this point, but requires much greater numbers. However, our results indicate that PBMCs collected from healthy volunteers of various ages were unaffected by pancratistatin. It is therefore unlikely that pancratistatin is toxic to a particular subtype of non-cancerous PBMC samples. The efficacy and selectivity of pancratistatin against clinical leukemia is based on 2 characteristic features of apoptosis, viz. nuclear morphology and lipid rearrangement within the plasma membrane. Our results suggest that pancratistatin could be a novel way to treat leukemia and may not cause adverse effects common to intravenous chemotherapy [5]. Paclitaxel has been used to treat many types of cancer, including leukemia, for over a decade; but it causes harsh side effects often associated with chemotherapy [6]. In contrast, pancratistatin is more effective against cancer cells than paclitaxel and has no significant effect on non-cancerous PBMC samples.

The mechanism of action and biochemical target of pancratistatin is currently being studied. Due to its broad-range effectiveness, we hypothesize that pancratistatin may selectively target cancer cell mitochondria, as opposed to a specific kinase or signalling protein. We have previously shown that pancratistatin causes mitochondrial membrane potential collapse and permeabilization, leading to reactive oxygen species (ROS) generation, cytochrome *c *leakage, caspase-3 activation, and apoptosis [1,3,7]. Recent reports indicate that some leukemia cells have increased basal levels of ROS, suggesting increased susceptibility to oxidative stress, which can lead to mitochondrial permeabilization and subsequent apoptosis [8]. It has also been reported that cancer cell mitochondria are dysfunctional and more susceptible to attack compared to non-cancerous cell mitochondria, which presents an opportunity for the development of new chemotherapeutics, especially for leukemia [9]. Liu *et al. *[10] reported that Lycorine, also an Amaryllidaceae alkaloid, induces caspase-dependent apoptosis in leukemia cells by down regulating the anti-apoptotic protein, Mcl-1. It is possible that pancratistatin is also targeting a protein that regulates the balance of apoptosis/survival, which could explain the broad-range efficacy of this compound against cancer cells *in vitro*. This is supported by our evaluation of pancratistatin on patient-obtained leukemia cells *ex vivo *at a low dose (1 μM).

## Conclusions

Our results show that pancratistatin is an effective and selective anti-cancer agent with potential for advancement to clinical trials [11]. Targeted therapies are the holy grail of systemic cancer therapy; the ideal drug should kill cancer cells without harming non-cancerous cells. This is an exciting development in the treatment of AML, for which treatment regimens used over the last 30 years have not significantly improved survival.

## Declaration of Competing interests

The authors declare that they have no competing interests.

## Authors' contributions

CG contributed to study design, data analysis and interpretation, and preparation of the manuscript. CH contributed to study design, data collection and interpretation. JM provided pancratistatin, and contributed to study design. SP contributed to study design, and data interpretation. All authors read and approved the final manuscript.
